# Autoinflammatory syndromes: report on three cases

**DOI:** 10.1590/S1516-31802009000500012

**Published:** 2010-02-03

**Authors:** Thais Cunha Matos, Maria Teresa Ramos Ascensão Terreri, Daniela Gerent Petry, Cássia Maria Barbosa, Claudio Arnaldo Len, Maria Odete Esteves Hilário

**Affiliations:** I MD. Pediatrician, Division of Pediatric Rheumatology, Department of Pediatrics, Universidade Federal de São Paulo (Unifesp), São Paulo, Brazil.; III MD, PhD. Assistant professor, Division of Pediatric Rheumatology, Department of Pediatrics, Universidade Federal de São Paulo (Unifesp), São Paulo, Brazil.; III MD. Attending physician, Division of Pediatric Rheumatology, Department of Pediatrics, Universidade Federal de São Paulo (Unifesp), São Paulo, Brazil.

**Keywords:** Relapsing fever, Familial mediterranean fever, Tumor necrosis factor-alpha, Child, NLRP3 protein, human [substance name], Inflammation, Febre recorrente, Febre familiar do mediterrâneo, Fator de necrose tumoral alfa, Criança, Inflamação

## Abstract

**CONTEXT:**

Autoinflammatory syndromes are diseases manifested by recurrent episodes of fever and inflammation in multiple organs. There is no production of autoantibodies, but interleukins play an important role and acute-phase reactants show abnormalities. Our aim was to report on three cases of autoinflammatory syndromes that are considered to be rare entities.

**CASE REPORTS:**

The authors describe the clinical features of three patients whose diagnosis were the following: tumor necrosis factor receptor-associated periodic syndrome (TRAPS), chronic infantile neurological cutaneous articular (CINCA) syndrome and familial Mediterranean fever (FMF). All of the patients presented fever, joint or bone involvement and increased acute phase reactants. The genetic analysis confirmed the diagnoses of two patients. The great diversity of manifestations and the difficulties in genetic analyses make the diagnosing of these diseases a challenge.

## RESUMO

**CONTEXTO:**

As síndromes auto-inflamatórias são doenças que se manifestam por surtos recorrentes de febre e inflamação em diversos órgãos. Não ocorre a formação de auto-anticorpos, as interleucinas representam um papel importante e as provas de fase aguda estão alteradas. O nosso objetivo foi relatar três casos de síndromes auto-inflamatórias consideradas como entidades raras.

**RELATO DE CASOS:**

Os autores descrevem as características clínicas de três pacientes que tiveram os seguintes diagnósticos: síndrome periódica associada ao receptor do fator de necrose tumoral a (TRAPS), síndrome articular cutânea neurológica e infantil crônica (CINCA) e febre familiar do Mediterrâneo (FFM). Todos os pacientes tiveram em comum a febre, o comprometimento articular ou ósseo e o aumento de provas de fase aguda. O estudo genético confirmou o diagnóstico em dois pacientes. A grande variedade de manifestações e a dificuldade nos estudos genéticos tornam o diagnóstico destas doenças um desafio.

## INTRODUCTION

Autoinflammatory syndromes are monogenic diseases characterized by episodes of recurrent systemic inflammation that usually present with fever, which may or may not be associated with cutaneous rash, serositis, lymphadenopathy or arthritis. Autoantibodies are not present, but cytokines play an important role and acute phase reactants show abnormalities. These syndromes are diseases of the innate immunity. Receptors present in this immunity, such as the nucleotide oligomerization domain (NOD) and pyrin domain-containing protein (NALP), which are responsible for recognition of pathogens or cell damage, can result in exaggerated inflammasome activation when mutated.^1^^,^^2^ Although most chronic or recurrent fevers within the pediatric age group are usually due to infection or neoplasia, autoinflammatory syndromes should also be considered, especially in cases of unknown origin. Included in these are the hereditary periodic fever syndromes: familial Mediterranean fever (FMF), tumor necrosis factor receptor-associated periodic syndrome (TRAPS), hyperimmunoglobulinemia D with periodic fever syndrome (HIDS) and cryopyrin-associated periodic syndrome (CAPS), which includes chronic infantile neurological cutaneous and articular (CINCA) syndrome, Muckle-Wells syndrome (MWS) and familial cold urticaria (FCU).

The aim of this study was to describe three patients with autoinflammatory syndromes who were attended in our Pediatric Rheumatology outpatient clinic, and to make clinicians aware of the diversity of manifestations and diagnostic complexity of these conditions. Table 1 contains a summary of the clinical and laboratory findings in the three cases.

**Table 1.** Clinical and laboratory findings from the three patients with autoinflammatory syndromes

	Case 1 TRAPS	Case 2 CINCA	Case 3 FMF
Fever	+	+	+
Cutaneous rash	+	+	-
Arthritis	+	-	+
Uveitis	-	+	-
Abdominal pain	+	-	+
Bone volume increase	-	+	-
Anemia	+	-	+
Acute-phase reactants abnormal	+	+	+
Family history	+	-	-
Genetic study confirmation	+	+	-

TRAPS = tumor necrosis factor receptor-associated periodic syndrome; CINCA = chronic infantile neurological cutaneous articular syndrome; FMF = familial Mediterranean fever.

## CASES REPORT

Case 1. A 23-year-old Caucasian boy presented at the age of nine years with fever (39 °C) associated with rash and cervical, abdominal and diffuse limb pain. He underwent laparoscopic surgery because of left flank pain, without findings. Over the next six years, he presented with recurrent crises of fever, thoracic pain, arthralgia, large joint arthritis and occasional skin rash. A diagnosis of systemic juvenile idiopathic arthritis (JIA) was made and the patient was treated with corticosteroids, chloroquine, indomethacin, sulfasalazine, methotrexate cyclosporine and gamma globulin. The laboratory tests revealed anemia, leukocytosis, thrombocytosis, erythrocyte sedimentation rate (ESR) of 120 mm in the first hour, C-reactive protein (CRP) of 47 mg/dl (normal £ 0.5) and ferritin level of 230 ng/ml (normal £ 140). The antinuclear antibody (ANA) showed a fine speckled pattern and a titer of 1/80. Use of anti-tumor necrosis factor (TNF) agents showed an excellent but transient response. Pulse therapy with methylprednisolone and oral corticosteroids were needed to control fever and pain. Questioned further, the parents mentioned a febrile illness associated with diffuse pain that the grandfather and great-grandfather had had. DNA analysis showed a mutation present in exons 2, 3 and 4 of the TNFRSF1A gene, thus resulting in a diagnosis of TRAPS. The patient’s illness worsened and he developed macrophage activation syndrome and kidney failure, leading to death.

**Figure 1.**
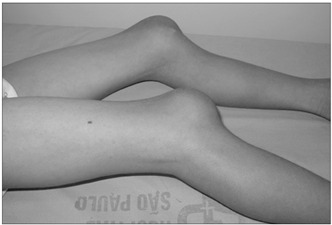
Increased bone volume in both knees (increased patellar volume) in patient 2 (chronic infantile neurological cutaneous articular syndrome, CINCA).

Case 2. An 11-year-old non-Caucasian boy complained of daily fever and non-pruritogenic rash since the age of two years. By the age of seven years, he also developed arthritis in both knees, in addition to the previous symptoms. At the age of 11 years, he presented persistent fever, maculopapular rash on the trunk and lower limbs, increased bone volume in both knees (increased patellar volume) (Figure 1) and acute anterior uveitis. He was also experiencing learning difficulties at school. No significant hemogram abnormalities were found. The laboratory findings included an ESR of 50 mm in the first hour and CRP of 4.0 mg/dl; ANA was negative and cerebrospinal fluid did not show any abnormalities. Radiographs on the lower limbs showed an increased volume of the epiphysis and metaphysis of the tibia and increased patellar bone volume. With these findings, CINCA syndrome was suspected. A genetic analysis on the patient showed a new mutation in the CIAS1 gene, thus confirming the diagnosis. The patient was treated with prednisone, indomethacin, methotrexate and anti-interleukin-1, with a partial response. At the present he still presents fever and rash but these manifestations are less frequent than at the disease onset.

Case 3. A 25-year-old Caucasian female of Italian origin had had symptoms beginning at the age of five years, including fever, localized abdominal pain, limb pain and arthritis, which occurred in crises that lasted a few days. Some episodes included pain in the diaphragmatic region. Physical examination revealed slight splenomegaly. Two years after the symptoms began, FMF was suspected, colchicine was introduced and these manifestations were controlled. The initial hemogram showed hemoglobin of 10.9 g/dl, hematocrit of 34%, normal leukocyte and platelet counts and an ESR of 56 mm in the first hour. An abdominal ultrasound and an echocardiogram were normal. Over the last 15 years, the patient has progressed well, but every attempt to withdraw colchicine has resulted in reemergence of the fever, abdominal and joint pain.

## DISCUSSION

Autoinflammatory syndromes are characterized by recurrent episodes of systemic inflammation and frequent fever.^3^ They are also characterized by the absence of any apparent infectious triggers, and flares are self-activated.

Although rare, they must be included in the differential diagnosis when dealing with fevers of unknown origin (whether recurrent or not). Family history and clinical features are important for the diagnosis, but confirmation is obtained through genetic analysis.

The characteristics that all of our cases had in common were fever, joint or bone involvement and elevated acute-phase reactants.

The patient in case 1 was thought to have had systemic JIA for a long time. The resemblance of the clinical manifestations and lack of knowledge of autoinflammatory syndromes resulted in a delay in diagnosis of 14 years. Failure to respond to therapy, good response only to very high doses of corticosteroids and the presence of a family history led us to suspect autoinflammatory syndrome.^4^ The use of anti-TNF resulted in clinical and laboratory remission of the disease, albeit transiently.

In case 2, the bone overgrowth alerted us to the possibility of CINCA, which was later confirmed through genetic studies. Lack of knowledge about this syndrome delayed the correct diagnosis by nine years.

In case 3, despite the fact that the ultrasound did not reveal ascites, the abdominal pain was interpreted as serositis, thus fulfilling the FMF triad (fever, arthritis and serositis). The Italian origin, recurrence of short-duration attacks and the excellent response to colchicine were also suggestive of the disease.^5^

The diagnosis of autoinflammatory syndromes must be suspected in cases that simulate systemic JIA. However, the family history and a poor response to treatment may suggest alternative diagnoses, which need to be confirmed through genetic testing. The presence of fever (whether recurrent or not), joint or bone involvement and abnormalities in acute-phase reactants are signs that should alert physicians to the possibility of this diagnosis. Lack of knowledge about these syndromes, difficulty in accessing genetic analyses and the limited biological therapies available (used in some cases) make these diseases a clinical challenge.

## CONCLUSION

Autoinflammatory syndromes should be considered in patients with fever of unknown origin and the clinicians must be aware of the diversity of manifestations and diagnostic complexity of these conditions.
